# Anatomical Footprint of the Tibialis Anterior Tendon: Surgical Implications for Foot and Ankle Reconstructions

**DOI:** 10.1155/2017/9542125

**Published:** 2017-06-04

**Authors:** Madeleine Willegger, Nargiz Seyidova, Reinhard Schuh, Reinhard Windhager, Lena Hirtler

**Affiliations:** ^1^Department of Orthopaedics, Medical University of Vienna, Vienna, Austria; ^2^Department of Plastic Surgery, Guy's and St. Thomas' NHS Foundation Trust, London, UK; ^3^Center for Anatomy and Cell Biology, Division of Anatomy, Medical University of Vienna, Vienna, Austria

## Abstract

This study aimed to analyze precisely the dimensions, shapes, and variations of the insertional footprints of the tibialis anterior tendon (TAT) at the medial cuneiform (MC) and first metatarsal (MT1) base. Forty-one formalin-fixed human cadaveric specimens were dissected. After preparation of the TAT footprint, standardized photographs were made and the following parameters were evaluated: the footprint length, width, area of insertion, dorsoplantar location, shape, and additional tendon slips. Twenty feet (48.8%) showed an equal insertion at the MC and MT1, another 20 feet (48.8%) had a wide insertion at the MC and a narrow insertion at the MT1, and 1 foot (2.4%) demonstrated a narrow insertion at the MC and a wide insertion at the MT1. Additional tendon slips inserting at the metatarsal shaft were found in two feet (4.8%). Regarding the dorsoplantar orientation, the footprints were located medial in 29 feet (70.7%) and medioplantar in 12 feet (29.3%). The most common shape at the MT1 base was the crescent type (75.6%) and the oval type at the MC (58.5%). The present study provided more detailed data on the dimensions and morphologic types of the tibialis anterior tendon footprint. The established anatomical data may allow for a safer surgical preparation and a more anatomical reconstruction.

## 1. Introduction

The tibialis anterior muscle is known as the strongest dorsal extensor of the foot and ankle. The muscle originates from the anterior-lateral surface of the tibia and continues to the dorsum of the foot where its tendon inserts at the base of the first metatarsal (MT1) and at the medial cuneiform (MC) [1–3]. A detailed understanding of the anatomy of the tibialis anterior tendon (TAT) insertion is crucial for surgical reconstruction as well as for tendon harvest in foot and ankle surgery. However, common anatomy textbooks provide a simplified description of the tendons bony insertion. Although several anatomical studies were focused on variations of distal TAT insertions, the precise position of the bony TAT footprint has received little attention [1, 2, 4–10].

Ruptures of the TAT are seldom conditions and can be caused by direct trauma or spontaneous rupture. Spontaneous ruptures happen predominantly on the basis of a degenerative process [9, 11–14]. Surgical reconstruction of the TAT is the treatment of choice in cases with severe impairment of dorsal extension and supination of the foot. Different techniques according to the severity of tendon injury or gap formation have been reported. In order to restore the natural lever arm of the tibialis anterior muscle, the tendon must be reinserted at its anatomical footprint [9, 11]. Precise anatomical description of ligament and tendon attachments is important and can help to optimize reconstruction procedures in terms of anchor placement or graft sizing.

The TAT also plays a major role in foot and ankle tendon transfers. Imbalance of neuromuscular function and residual dynamic clubfoot deformity in children are common indications for TAT or split TAT transfers. Surgical preparation of the tendon at its insertion makes the knowledge of the anatomical course and anatomy compulsory [15–17].

This study aimed to analyze precisely the insertional footprints of the TAT (tibialis anterior tendon) and its variations. It was anticipated that this information would aid in performing an anatomical reconstruction and thus clarifying the local anatomical prerequisites for tendon harvest.

## 2. Materials and Methods

Forty-one (41) adult formalin-fixed lower leg specimens were included in this study. The specimens were obtained from voluntary donors who consented during lifetime to donate their body for research and teaching purpose to the Center for Anatomy and Cell Biology at our Medical University. The study has been approved by the local ethics committee (EK 1555/2015).

The mean age of the 26 female and 15 male donors was 85.2 years (67–101). Twenty (20) left and twenty-one (21) right lower legs were dissected. Inclusion criteria were sufficient quality of the specimen and no evidence of surgical intervention in the area examined, to allow for a complete identification of the tendon attachment. The skin, subcutaneous tissue and all muscles except for the tibialis anterior muscle were removed from the lower legs with a scalpel. Care was taken not to injure any ligamentous structure especially the tibialis anterior tendon (TAT) and its corresponding bony insertions. Each course of the TAT was documented by photograph according to a standardized protocol. Following exposure of the bony attachments of the TAT, the tendon was carefully dissected and removed at the bony insertion. Its “footprint” was marked with ink and documented by photograph with a ruler in a standardized manner. A split tendon or additional variations of the tendinous extension slips were evaluated descriptively. In order to identify the precise extent of the footprint, the bones of the midfoot and hindfoot were disarticulated and again photographed with a reference ruler.

**Figure 1:**
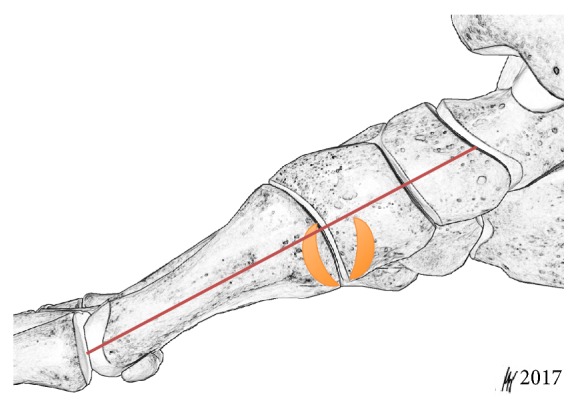
For evaluation of the dorsoplantar location of the TAT footprint, the longitudinal axis of the first metatarsal bone has been drawn as a reference line. Footprints which crossed the line were classified as medial and footprints located plantar to the reference line were rated medioplantar. The schematic drawing depicts a medial MT1 footprint insertion with a crescent shape.

Measurements of the footprint dimensions (length and width, mm) were conducted and areas of insertion (AOI, mm^2^) were calculated. All photos were digitally measured by use of Image J (http://rsb.info.nih.gov/ij/) software [18]. Different types of TAT footprints were distinguished according to the shape and area of the tendon attachment. In order to clarify which bony insertion contributes more to the TAT footprint, the Musial classification has been used. The classification defines an equal footprint at the MC and MT1, a wide insertion at the MC and narrow insertion at the MT1, and a narrow insertion at the MC and wide insertion at the MT1 or a principal insertion at the MC and some accessory slips at MT1 [2]. To define a dorsoplantar location of the footprints at the medial aspect of the corresponding bones, the longitudinal axis of the first metatarsal was drawn as reference line. The entire insertion at the MC and MT1 was taken into account. Footprints located plantar to that line were defined as medioplantar. If a footprint crossed or touched that line, the location was specified as medial (Figure 1). Area and distance measurements are reported as averages with the range.

## 3. Results

**Figure 2:**
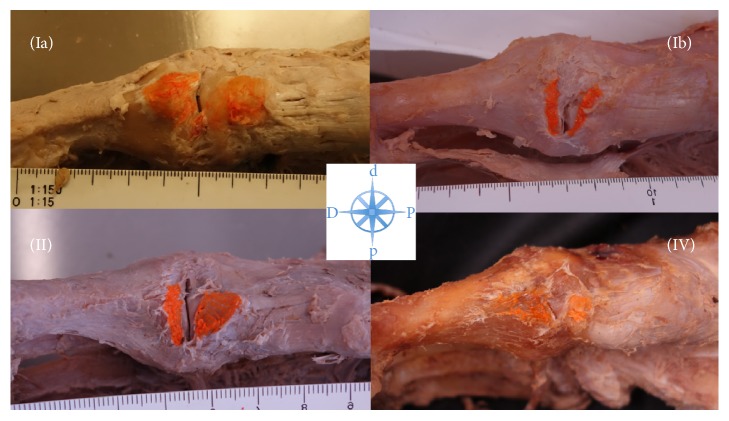
The different types of TAT insertions. Standardized photographs of right feet from a medial view show the TAT footprints after marking with orange ink. D = distal; P = proximal; d = dorsal; p = plantar.

The TAT inserted in all 41 specimens (100%) at the first metatarsal base and the medial cuneiform. According to the proposed classification by Musial [2], 20 feet (48.8%) showed a Type I insertion (equal insertion at the MC and MT1), 20 feet (48.8%) showed a Type II insertion (wide insertion at the MC and narrow insertion at the MT1), and 1 foot (2.4%) showed a Type IV insertion (narrow insertion at the MC and wide insertion at the MT1), respectively. Due to the heterogenic morphological appearance of the TAT footprint in Type I, we further subclassified Type I into Ia (wide insertion at the MC and MT1) and Ib (narrow insertion at the MC and MT1). Type Ia was identified in 3 feet (7.3%) and Type Ib in 17 feet (41.5%) (Table 1 and Figure 2). None of the specimens showed a Type III (principal insertion at the MC and some accessory slips at MT1) insertion.

The mean width of the TAT footprint at the MC was 6.7 mm (range 2.0–14.4) and the mean length was 13.9 mm (range 8.4–22.6). For the MT1 footprint the corresponding sizes were 4.6 mm (range 1.6–14.7) and 14.0 mm (range 9.2–20.2), respectively. At the medial cuneiform the mean area of insertion (AOI) revealed 71.5 mm^2^ with a range from 20.1 to 151.0 mm. The mean footprint area at the first metatarsal base was measured 48.1 mm^2^ (range 18.5–97.0). The footprint of the MC represented 59.8% of the area of insertion of the conjoined ATT attachment. With regard to the dorsoplantar orientation, the footprints were located medial in 29 feet (70.7%) and medioplantar in 12 feet (29.3%) (Figure 1).

**Figure 3:**
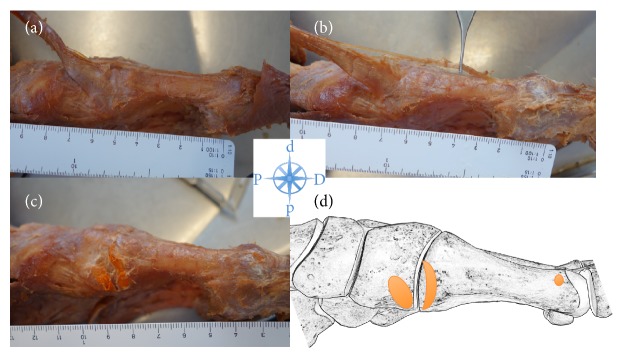
Specimen of a left foot with an additional tendon slip inserting at the distal first metatarsal shaft (variant). (a)-(b) show the tibialis anterior tendon with the variant tendon slip; (c) shows the footprints of the TAT after dissection of the tendon; (d) outlines the footprints in a schematic drawing.

Additional tendon slips were found in two feet (4.8%). One slip inserted at the proximal first metatarsal shaft and one at the distal metatarsal shaft (Figure 3).

The morphological shapes of the footprints were classified as oval, crescent, or triangular. The most common shape at the MT1 base was the crescent type (75.6%) and at the MC it was the oval type (58.5%) (Table 2). A subtendinous bursa of the ATT tendon was found in 8 feet (19.5%).

## 4. Discussion

Precise anatomical description of the insertion and footprint of the tibialis anterior tendon facilitates a safe surgical preparation and anatomical reconstruction. In our series of 41 feet we found a split TAT footprint with an insertion at the first metatarsal base and at the medial cuneiform in all dissected specimens. The larger footprint with a corresponding mean area of insertion of 71 mm^2^ (59.8% of the overall area of the footprint) is located at the medial cuneiform. Topographic evaluation revealed a medial located footprint in 70% and a medioplantar footprint in approximately 30% of feet. We defined different types of insertions according to the size and shape of the corresponding areas of insertion. The majority of specimens showed a larger footprint at the medial cuneiform. Additional tendon slips were found in 2 specimens (4.8%).

According to the original classification of TAT insertions by Musial [2], there exist 4 different types: Type I, with an equal insertion at the MC and first MT; Type II, with a wide insertion at the MC and a narrow insertion at the first MT, Type III, with a principal insertion at the MC and only a small tendon slip at the first MT; and Type IV, with a wide insertion at the first MT and a narrow insertion at the MC. In the present study the classification has been modified with a subclassification of Type I into Ia (wide insertion at the MC and MT1) and Ib (narrow insertion at the MC and MT1). Type Ib (41.5%) and Type II (48.8%) were the most common insertion patterns in our series.

Brenner dissected 156 feet looking for differences in the TAT insertion between normal feet and feet with hallux valgus deformity. Differences between hallux valgus feet and normal feet could not be determined in this study. Regarding the insertion sites he found 3 feet (1.9%) with a single insertion at the first metatarsal base and 2 feet (1.3%) with an insertion at the medial cuneiform, respectively. The majority of specimens showed an insertion on both bones (96.2%) [1].

In another study, Anagnostakos et al. reported the insertion of the tibialis anterior tendon in 53 feet. They found 68% with an attachment at the medial cuneiform and the first metatarsal base. 25% of feet showed a single footprint at the medial cuneiform but no specimen was found with an insertion at the first metatarsal base only. In contrast, our study population showed footprints on both bones in all dissected specimens (100%). This difference might be due to the smaller sample size in the present study. Synoptically our results correspond well with the findings of Brenner [1] and Musial [2]. A subtendinous bursa has been detected in 17.3% of cases by Brenner [1]. Our study confirms the presence of a bursa in 19.5% of specimens.

Tibialis anterior tendon ruptures are an uncommon pathology, but case reports and series of surgical reconstructions are increasing since its first description in 1905 [19]. Particularly patients who experience a major loss of ankle dorsiflexion and foot supination strength accompanied by gait disorders with a steppage gait, or foot-slapping, benefit from surgical repair. There is also a trend for primary surgical repair in nontraumatic degenerative ruptures. Different techniques have been described [9, 11, 14, 20–23]. Anatomic reconstruction of the natural course and biomechanical lever arm should be pursued in order to restore dorsal extension power and forefoot supination. Tendon to bone reattachment is usually performed by use of bone anchors which should be placed at the anatomical insertion. In cases with a retracted tendon which cannot be apposed onto its insertion site, an interpositional tendon autograft or allograft can be used for reconstruction or augmentation. Knowledge of the size and location of the footprint is useful in surgical decision making [24–26].

Tendon transfers around the foot are commonly used surgical procedures for balancing or tethering the motion of the foot and ankle during gait. Indications for TAT transfer range from dynamic clubfoot residual or spastic deformities in children to an impaired peroneal tendon function in adults. The accurate description of TAT insertion may assist surgeons during preparation and tendon harvest [15, 17, 27].

The close relation of the TAT to the first tarsometatarsal joint (TMTJ) is also a focus in hallux valgus surgery. First TMTJ arthrodesis with plate fixation is a popular surgical procedure due to a powerful angular correction potential [28]. Recent biomechanical and anatomical evidence suggests the use of plantar plates. Plaass et al. [29] defined a safe zone for plantar plate placement in first TMTJ arthrodesis by outlining the attachment of the tibialis anterior and the peroneus longus tendon. Their study further showed that plate design according to anatomical prerequisites is essential in aiming for preservation of tendon attachments. Strict plantar placement of a plate does not interfere with the tibialis anterior tendon in first TMTJ/Lapidus arthrodesis.

It is acknowledged that this study comprises some limitations. Described differences in anatomical insertion of the TAT may vary due to the geographical origin and the number of examined specimens. Therefore the measured anatomical footprints may not be representative of the general population. A detailed analysis regarding the differences between male and female footprint variations was omitted due to the sample size. Nevertheless, the relative small sample size of 41 dissected feet is still an acceptable quantity for a study with anatomical specimen.

The present study provided more detailed data on the dimensions and morphologic types of the tibialis anterior tendon footprint. The different shapes and topographic locations have been described for the first time. However, the newly gained information can help in surgical preparation and may enhance further development of new surgical techniques for tibialis anterior tendon reconstruction.

## 5. Conclusion

This study provides a comprehensive qualitative and quantitative anatomical analysis of the insertion of the tibialis anterior tendon. The present data will enhance the current knowledge on the anatomy of the TAT footprint and can be used as reference for anatomical reconstructions or subsequently assist in surgical preparation for tendon harvest.

## Figures and Tables

**Table 1:** Tibialis anterior tendon insertions according to the modified classification by Musial [2].

Type	MT1 footprint	MC footprint	Feet (%)
Ia	Wide	Wide	3 (7.3%)
Ib	Narrow	Narrow	17 (41.5%)
II	Narrow	Wide	20 (48.8%)
III	Slips	Wide	0 (0%)
IV	Wide	Narrow	1 (2.4%)

**Table 2:** Distribution of different shape patterns of the tibialis anterior tendon footprint.

Shape	MT1 (feet (%))	MC (feet (%))
Oval	8 (19.5%)	24 (58.5%)
Crescent	31 (75.6%)	10 (24.4%)
Triangular	2 (4.9%)	7 (17.1%)
